# Comparative Investigation on Improved Aerodynamic and Acoustic Performance of Abnormal Rotors by Bionic Edge Design and Rational Material Selection

**DOI:** 10.3390/polym14132552

**Published:** 2022-06-23

**Authors:** Wenda Song, Zhengzhi Mu, Yufei Wang, Zhiyan Zhang, Shuang Zhang, Ze Wang, Bo Li, Junqiu Zhang, Shichao Niu, Zhiwu Han, Luquan Ren

**Affiliations:** 1 Key Laboratory of Bionic Engineering of Ministry of Education, Jilin University, Changchun 130022, China; songwd21@mails.jlu.edu.cn (W.S.); yufeiw21@mails.jlu.edu.cn (Y.W.); zzytt1126@163.com (Z.Z.); zhangshuang20@mails.jlu.edu.cn (S.Z.); wangze@jlu.edu.cn (Z.W.); boli@jlu.edu.cn (B.L.); junqiuzhang@jlu.edu.cn (J.Z.); niushichao@jlu.edu.cn (S.N.); zwhan@jlu.edu.cn (Z.H.); lqren@jlu.edu.cn (L.R.); 2 School of Mechanical and Aerospace Engineering, Jilin University, Changchun 130022, China

**Keywords:** abnormal rotor, bionic design, aerodynamic performance, noise reduction, UAV

## Abstract

Rotor plays a vital role in the dynamical system of an unmanned aerial vehicle (UAV). Prominent aerodynamic and acoustic performance are a long-term pursuit for the rotor. Inspired by excellent quiet flight characteristics of owls, this work adopted bionic edge design and rational material selection strategy to improve aerodynamic and acoustic performance of the rotor. A reference model of rotor prototype with streamlined edges was firstly generated by reverse engineering method. With inspiration from owl wings and feathers, bionic rotors with rational design on leading and trailing edges were obtained. Original and bionic rotors were fabricated with polyamide PA 12 and Resin 9400 by 3D printing technique. Aerodynamic and acoustic performance of the as-fabricated rotors were experimentally measured and analyzed in detail using a self-established test system. Comparative experimental results indicated that the aerodynamic and acoustic performance of the rotors was closely related to the bionic structures, material properties, and rotational speeds. At the same rotational speed, bionic rotor fabricated with Resin 9400 can produce a higher thrust than the prototype one and its power consumption was also reduced. The resulting noise of different bionic rotors and their directivities were comparatively investigated. The results verified the bionic edge design strategy can effectively control the turbulent flow field and smoothly decompose the airflow near the tailing edge, which resulting in enhancing the thrust and reducing the noise. This work could provide beneficial inspiration and strong clues for mechanical engineers and material scientists to design new abnormal rotors with promising aerodynamic and acoustic performance.

## 1. Introduction

In recent years, the multi-rotor UAV has been widely used in various fields due to their unparalleled maneuverability, mechanical reliability, and high security. [1]. In civilian applications, multi-rotor UAV becomes the first choice for aerial photography and delivery due to their low price and ease of operation [2]. In military applications, multi-rotor UAV is usually used to complete search and reconnaissance missions [3], as well as possible UAV swarm combat missions in the future [4]. In addition, multi-rotor UAV is also widely used in the agricultural field [5]. The multi-rotor UAV has also been used in the study of volcanic gas plumes because of its stable flight in complex environments. [6]. With the rapid development of the multi-rotor UAV, two prominent issues, including cruising ability [7] and environmental protection [8], have become more and more urgent. These problems need to be addressed and could be resolved by improving aerodynamic performance and reducing noise pollution. Learning from the miraculous nature, scientists and engineers have constantly devoted themselves to seeking satisfactory solutions to these problems [9,10,11,12,13,14,15,16].

Owls as star raptors have been intensively investigated due to their striking ability to fly silently and capture large prey. It was found that owls’ unique feather structures are the key factors for its excellent flight ability. Generally, its feathers are normally characterized by leading edge combs, trailing edge fringes, velvet-like surfaces, and directional textures [17,18,19]. The raised structures on the leading edge of the wing were considered to improve the aerodynamic characteristics and increase the lift of the wings, which could contribute to the maneuverability of the owl [20,21,22]. It is worth mentioning that this leading edge structure was also found on the flippers of a humpback whale, which can improve the attack angle before stall occurs [23]. Trailing edge fringes of owl feathers can significantly reduce scattering noise from the trailing edge [24]. Similarly, leading edge combs can reduce turbulence intensity across the trailing edge [25]. Distal barbules of feathers intersperse with each other to form a multi-layer porous structure. Velvet-like structures on the wing surface can soften airflow and reduce boundary layer turbulence [21]. In addition, noise reduction can also be realized through directional textures formed by interlaced feathers of owl’s wings. When airflow passes through the wing surface, the directional textures can effectively make the airflow adhere to the wing surface and restrain the airflow separation to form a large eddy current, resulting in noise reduction [19].

Inspired by these promising characteristics of owl wings, a great deal of studies try to reduce noise and improve aerodynamic performance of rotors by elaborate morphology design. Hu et al. [26] designed leading edge with serrations to investigate the influence of different serration parameters, including height and wavelength on noise reduction performance. The maximum noise reduction was up to 13 dB, while the aerodynamic performance of the rotor also decreased. Liu et al. [27] added ridge structures to the leading edge, which also demonstrated good noise reduction effect. It was proved by numerical simulation that bionic structures can cause changes of streamwise vortices. Besides, the influence of bionic trailing edge fringes on the noise and thrust performance of rotors was also investigated and discussed [8,28,29]. Candeloro et al. [30] experimentally confirmed that a sensible noise reduction with strong directivity can be achieved. Despite this, the reduction of thrust coefficient cannot be ignored. Most recently, Chen et al. [31] demonstrated bionic design of wind turbine blades that coupled owl wing shape with herringbone groove structures of feathers together. The herringbone groove structures on the surface of wind turbine blades enhanced flow adhesion by generating eddy currents and reduced pressure on the leeward side of the bionic blade. Previous studies suggested that leading edge serrations and trailing edge fringes are both effective structures for noise reduction. However, in most cases, the designs of leading edges and trailing edges were applied separately. Few studies considered these bionic structures at the same time. Herein, with inspiration from owl wings and feathers, bionic edge design was applied on abnormal rotors with raised structures on leading edge and the serrated structures on trailing edge. The aerodynamic and acoustic tests of original and bionic rotors were conducted under laboratory conditions. The experimental results were compared and discussed in detail to reveal the underlying work mechanism of the bionic abnormal rotors. This work is anticipated to inspire novel design and propose referable paradigm for abnormal rotors of small UAV, even turbine blades of large aeromotors.

## 2. Materials and Methods

### 2.1. Vital Structure Parameters of Original Rotor

**Figure 1:**
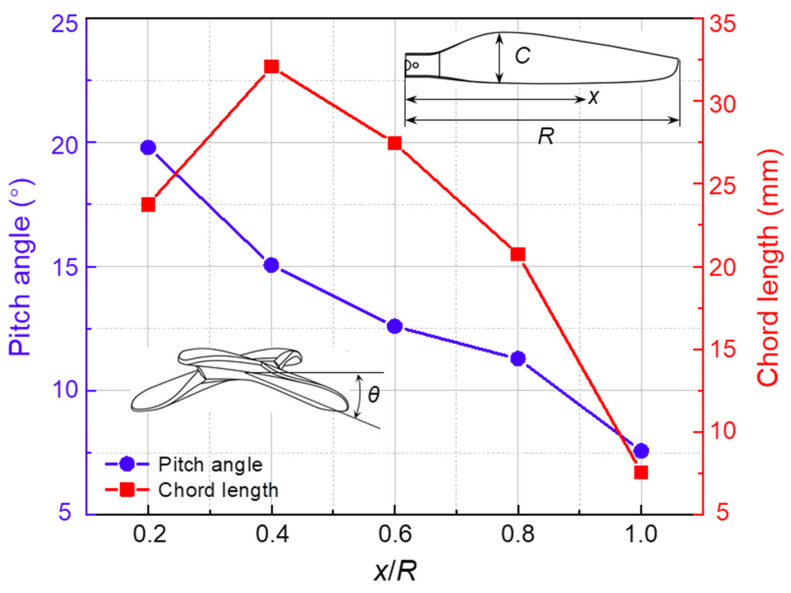
The radial distributions of the chord length and pitch angle of the OR rotor. Inset: Definition of variables *C*, *x*, *R*, and *θ*.

A commercial P-series carbon fiber (CF) rotor for small multirotor UAV was purchased from T-MOTOR company (Jiangxi Province, China), which was taken as the original rotor (OR). The OR is made of carbon fiber reinforced polymer (CFRP) and its weight is 14.2 ± 1.5 g. The geometry parameter of this OR is 13 × 4.4 inch (diameter × pitch). The spanwise distribution of chord length (*C*) and pitch angle (*θ*) of each section is shown in Figure 1. The chord length ranges from 7.6 to 32.1 mm. In order to describe the parameters of different positions of the rotor, the radius of the rotor is defined as *R*, the spanwise distance from any point on the rotor to the center of rotation is defined as *x*, and *x*/*R* is used to represent the position of different sections. Along the spanwise direction, it reaches the maximum value (32.1 mm) at the position of 0.4 times the radius (0.4*R*) near the OR root and reaches the smallest value (7.6 mm) at the OR tip. The pitch angle ranges from 7.6° to 19.8°. Similarly, it reaches the largest value (19.8°) at 0.2*R* near the OR root and gradually decreases along the spanwise direction to reach the smallest value (7.6°) near the OR tip. The maximum speed (υ) of the OR cannot exceed 8000 rpm and the ultimate tensile force (*F*) can reach 4.5 kg. In addition, considering the aerodynamic characteristics of the OR at different positions, the chord length at 0.75*R* is defined as the characteristic chord length (*C*_0_ = 22.3 mm) of the OR. It was used as an important reference parameter for the bionic design of leading edge and trailing edge.

### 2.2. 3D Reconstruction Model of OR

**Figure 2:**
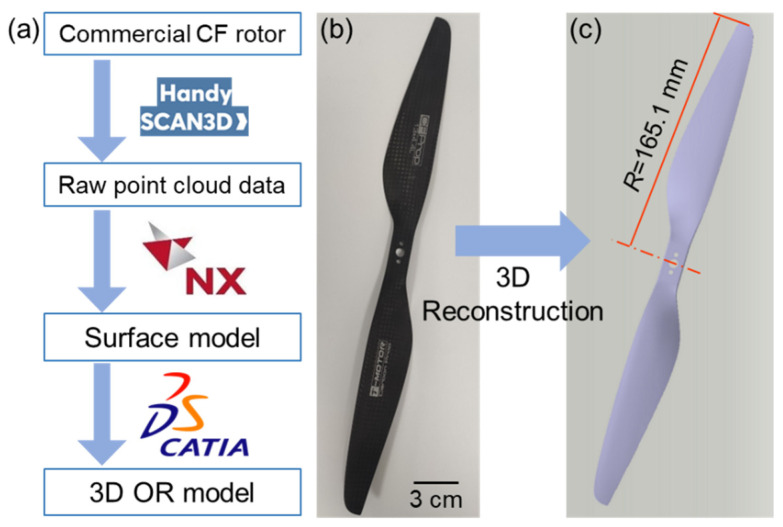
3D reconstruction process. (**a**) Workflow of 3D reconstruction model of OR via inverse engineering method; (**b**) A commercial CF rotor; (**c**) The obtained 3D reconstruction model of the OR.

The complete 3D reconstruction workflow is shown intuitively for better understanding of the reconstruction process (Figure 2a). Since the actual OR rotor is featured with typical complex overflow surfaces (Figure 2b), it is technically difficult to build an applicable reference model of the OR directly. In this case, inverse engineering method was adopted to construct a 3D model of the OR for following bionic edge design. First, a handheld 3D scanner (Handyscan 370, Creaform Shanghai Ltd., China) was used to obtain the raw point cloud data of the OR with a high-level accuracy of 0.03 mm. Second, the point cloud data was imported into an inverse modeling software (Unigraphics NX). Then, point cloud reconstruction, repair, and a series of post-processing were carried out to obtain a suitable rotor surface model with an accuracy of 0.10 mm. Finally, the obtained rotor surface model was imported into a commercial 3D modeling software (CATIA) to obtain 3D reconstruction model of OR (Figure 2c).

### 2.3. Structure Acquisition from Owl and Bionic Edge Design

**Figure 3:**
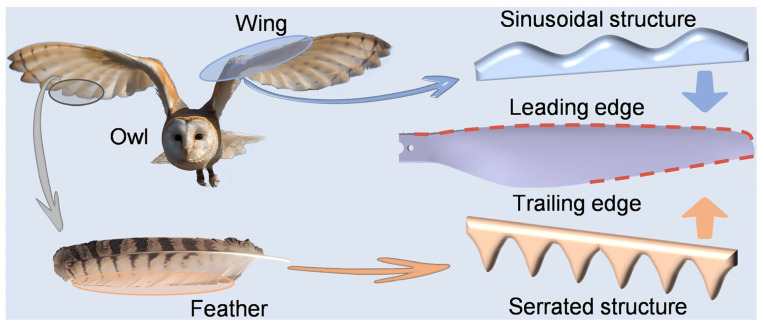
Bionic edge design strategy for leading and trailing edges of the OR with inspiration from owl wings and feathers. Source of the owl image: https://pixy.org/4654605 (accessed on 20 March 2022), reproduced with permission.

Both leading edge comb of owl wings and trailing edge fringes of its feathers both play a non-negligible role in owl flight. Inspired by this, bionic edge design for leading and trailing edges of the rotor was performed by acquiring design elements from owl wings and feathers (Figure 3).

**Figure 4:**
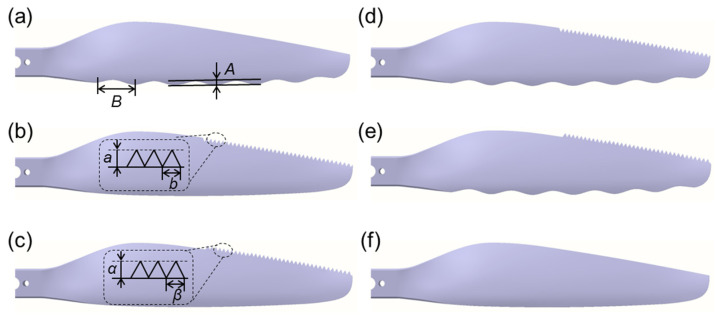
Planforms of different rotor models. (**a**) LE; (**b**) TE-S; (**c**) TE-A; (**d**) LE-TE-S; (**e**) LE-TE-A; (**f**) OR. Note: Since the rotor is a rotationally symmetric model, only half of each rotor is shown.

In fact, sinusoidal structures were widely studied for leading edge design and proved to be effective in many cases [26,27,32]. In this case, the sinusoidal structure acquired from owl wings was reasonably modified and streamlined to reduce aerodynamic loss of the rotor. For trailing edge design, it confirmed that serrated structures can suppress aerodynamic noise generated by high-speed rotation of the rotor [33,34]. Interestingly, the fringes in owl feathers possessed similar natural serrated structures, which was more production-friendly than conventional sinusoidal type [35,36]. During continuous rotation of the rotor, the linear velocity, vortex intensity, and turbulence near rotor tip are much larger than that near rotor root. To observe more remarkable acoustic performance, bionic serrations were designed and applied on the trailing edge near the rotor tip, which was along the span of 0.6~1.0 times the rotor radius [26,37]. The bionic serrations in this case are also set within the above-mentioned rotor area. Here, two design methods, including subtractive material design (SMD) and additive material design (AMD), were adopted to modify trailing edges of the bionic rotors. Therefore, a series rotor model with bionic edges was design (Table 1). According to the number of bionic edges, the rotor models can be divided into three groups: (1) Rotor with bionic leading edge (LE, Figure 4a), rotor with bionic trailing edge by SMD (TE-S, Figure 4b), and rotor with bionic trailing edge by AMD (TE-A, Figure 4c); (2) Rotor with both bionic leading edge and trailing edge by SMD (LE-TE-S, Figure 4d), rotor with both bionic leading edge and trailing edge by AMD (LE-TE-A, Figure 4e); (3) OR without bionic edge (OR, Figure 4f). It should be pointed out that the accuracy of the 3D printing used in this case is within 0.2 mm, which is much smaller than the feature size of bionic structures. Thus, the surface roughness will not significantly affect the flow distribution around the bionic rotors. In addition, the volumes of different rotor models were also provided to better describe the physical nature of these rotors (Table 2).

### 2.4. 3D Printing Rotors with Different Materials

**Figure 5:**
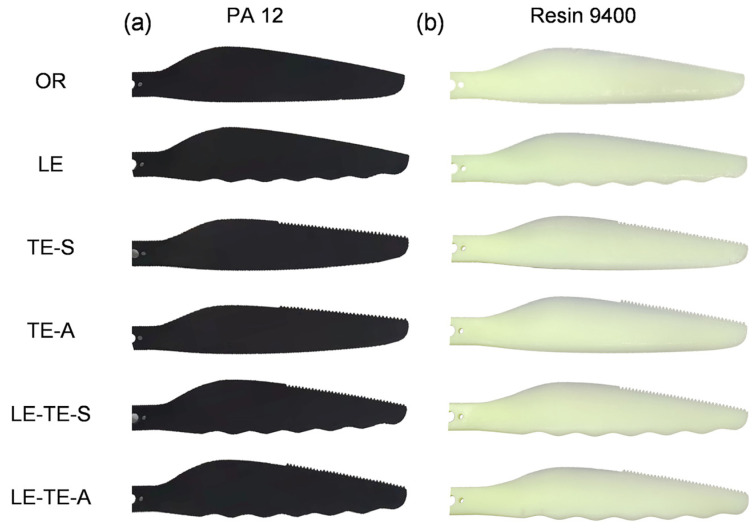
Different 3D printing rotors with PA 12 and Resin 9400, respectively. (**a**) OR and bionic rotors with PA 12; (**b**) OR and bionic rotors with Resin 9400.

3D printing technique with facile material selection has been wildly applied to the manufacture of complex structures. To introduce the abnormal bionic edges as designed above, a 3D printing technique was adopted to manufacture both OR and bionic rotors. It was well-known that photosensitive resin was one of the most commonly used materials for conventional 3D printing. However, when compared with the commercial CF rotor, the mechanical properties of rotors with photosensitive resin materials are far from enough. CFRP seemed to be a promising choice to manufacture bionic rotors with competitive mechanical properties. Unfortunately, the surface quality of CF rotors manufactured by 3D printing was hard to be satisfactory. It was hard to meet the relatively strict quality requirements of rotor surface to perform the following aerodynamic tests. Based on this factor, two common commercial materials for 3D printing that meet both strength requirements and have different properties were selected to fabricate the rotors (Table 3). According to as-built rotor models, twelve 3D printing rotors with PA 12 (Dongguan Zhonger New Material Co., Guangdong Province, China) and Resin 9400 (Dongguan Aide Polymer Material Technology Co., Ltd., Guangdong Province, China) were obtained (Figure 5). In addition, weight information of these rotors was also provided (Table 4).

### 2.5. Experimental Test Setup for Aerodynamic and Acoustic Performance

**Figure 6:**
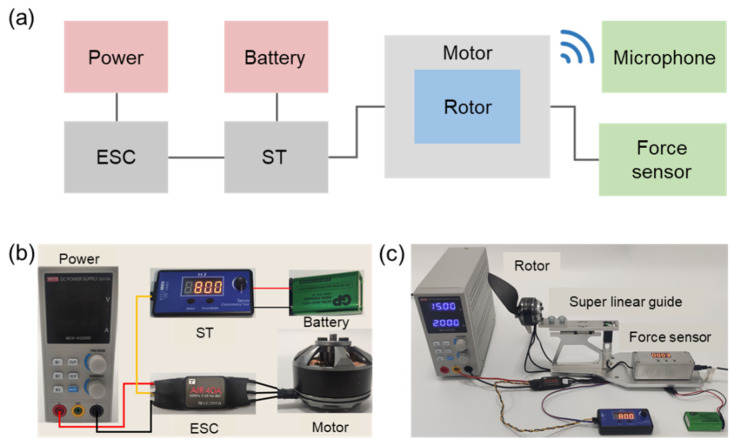
Experimental test setup. (**a**) Workflow of the test setup for aerodynamic and acoustic performance; (**b**) The connection state of the main components in the DTS; (**c**) DTS assembled with the RTTP at the stand-by state.

To reflect as much as possible the influence of the bionic structure on the rotors’ performance under real working conditions, the following experimental tests on aerodynamic and acoustic performance were conducted in an open space using a self-built difunctional test system (DTS). In this DTS, a small multi-axis multi-rotor power motor (T Motor MN4010 series, Nanchang, China) was used as the drive motor for the rotor. Then, the rotor was installed on the motor for debugging. A square wave brushless electronic speed control (ESC, T Motor AIR 20A, Nanchang, China) and servo tester (ST, T Moter MN4010, Nanchang, China) were selected as the control devices. An adjustable power supply (MCH-K3205D, Shenzhen Meichuang Instrument Co., Shenzhen, China) and a rechargeable battery (1604G 6F22 9V, Panasonic, Japan) were used to power the motor and ESC, respectively. After connecting all the devices and debugging, the motor speed can be adjusted by ST from 3000 rpm to 6000 rpm. The whole test system, including the above-mentioned components, is shown in Figure 6. For aerodynamic performance tests, a horizontal rotor tension test platform (RTTP) was selected. The RTTP consisted of a high-precision linear guide and a tension force sensor with a response range of 0–10 kg and an accuracy of 1 g. The horizontal RTTP can effectively eliminate the weight influence of the motor and accurately measure the pulling force of the rotor. In addition, the power consumed by the rotor can be calculated from the output voltage and current of the adjustable power supply.

**Figure 7:**
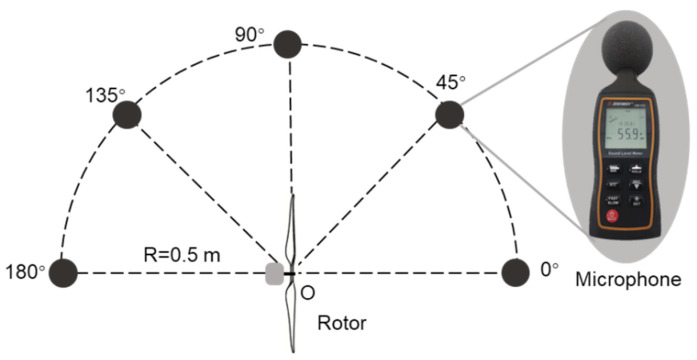
Schematic illustration of semicircular microphone array for acoustic performance test.

For acoustic performance tests, professional microphone array (SW-524, Sndway, Guangdong Province, China) was placed along a semicircular locus with a diameter of 0.5 m at an interval of 45°. The center of the microphone was at the same height as the rotation center of the rotor (Figure 7). The microphone accuracy was 0.1 dB and the noise data was collected by the NoisMeter 1.0 software. The aerodynamic and acoustic performance of all kinds of rotors were tested at a speed interval of 600 rpm in the range of 3000–6000 rpm. Speed calibration of the rotor was performed by a laser non-contact tachometer (TA8146C, Suzhou Tasi Electronics Co., Ltd., Jiangsu Province, China). Experimental data was collected and analyzed to compare aerodynamic and acoustic performance of rotors with different edges. In addition, the average background noise of different motor speeds in the open space was measured to more intuitively compare the noise reduction effects of different bionic rotors (Table 5). The noise levels of different rotors below are all measured under the corresponding background noise.

## 3. Results and Discussion

### 3.1. Thrust Generation

As a vital performance index of the dynamical system of an UAV, aerodynamic properties of the rotor have always been the focus of attention in the field of mechanical and aerospace engineering, which also promotes the booming development of various multi-rotor aircraft. Importantly, aerodynamic performance of the rotor directly affects endurance time and maximum take-off weight of the multi-rotor aircraft. Therefore, key indexes, including thrust and power consumption, were investigated to evaluate the aerodynamic performance of different rotors with bionic edges.

**Figure 8:**
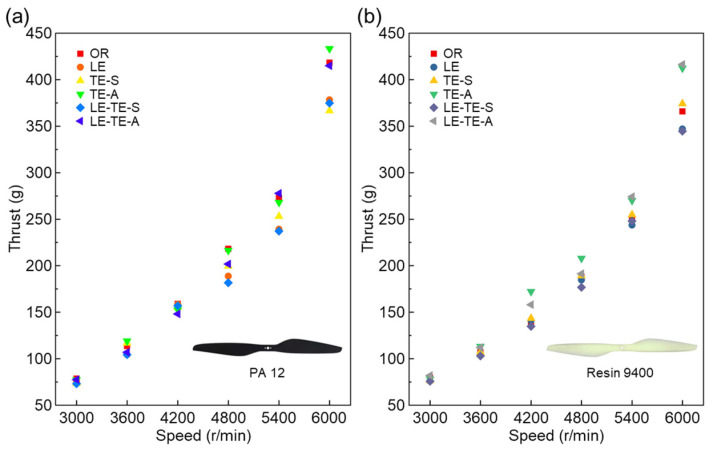
Thrust performance with different rotors. (**a**) Thrust performance of bionic rotors with PA 12; (**b**) Thrust performance of bionic rotors with Resin 9400.

For both PA 12 and Resin 9400 rotors, with rotation speed increasing from 3000 rpm to 6000 rpm, the average thrusts of all rotors demonstrated clear upward trends (Figure 8). The results were consistent with empirical cognition. Particularly, the thrust values showed a more significant increase after rotation speed reached 4800 rpm. It indicated that the thrusts of all rotors could be improved at a relatively high speed. Interestingly, when compared with different rotors at the same speed condition, most 3D printing rotors with resin 9400 showed decreased phenomena on thrust. The decrease phenomenon was more pronounced at a high rotation speed. This could be attributed to different mechanical properties of PA 12 and Resin 9400. Excellent mechanical properties can prevent rotor from edge warping or destructive deformation even at high rotation speeds, and vice versa. It found that most bionic rotors with PA12 (Figure 8a) and Resin 9400 (Figure 8b) can achieve similar thrust at lower speeds (3000–3600 rpm). More importantly, the bionic rotors TE-A with PA 12 showed the best thrust (433.4 ± 7.9 g) at high speed (6000 rpm).

**Figure 9:**
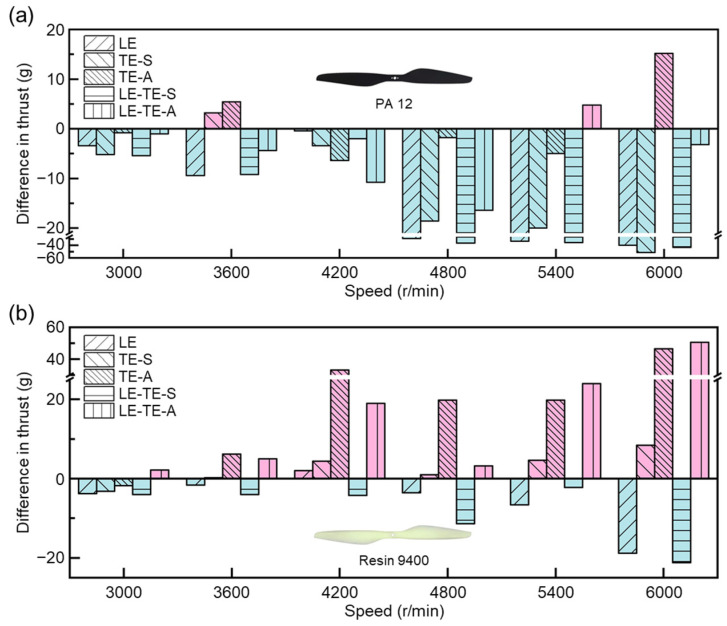
Comparison of thrust with different rotors. (**a**) Bionic rotors with PA 12; (**b**) Bionic rotors with Resin 9400. The thrust of the OR rotor was taken as a reference value (0 g). Under the same conditions, the increase in thrust was positive and the decrease in thrust was negative.

Taking the OR rotor as a reference, the effects of different bionic edge designs on aerodynamic performance are summarized (Figure 9). In contrast, it was also found that the sinusoidal structure applied on the leading edge will partially reduce the thrust of bionic rotors LE with PA 12 and Resin 9400, respectively. This is possibly due to the disturbance of flow distribution that arose by the sinusoidal structure near the leading edge [38]. Further, a more notable drop in the thrust of bionic rotor LE-TE-S with Resin 9400 can be found, resulting from the reduction of total effective lifting area by SMD, while the bionic rotor LE-TE-A with Resin 9400 showed a significant increase thrust during the whole speed range (3000–6000 rpm). Particularly, it can boost thrust by 13.83% when compared with OR with Resin 9400 at 6000 rpm. It provided evidence that the existence of TE-A can effectively control the turbulent flow near the leading edge, thus improving thrust. These results confirmed the validation of bionic edge design for aerodynamic performance improvement.

### 3.2. Power Consumption

**Figure 10:**
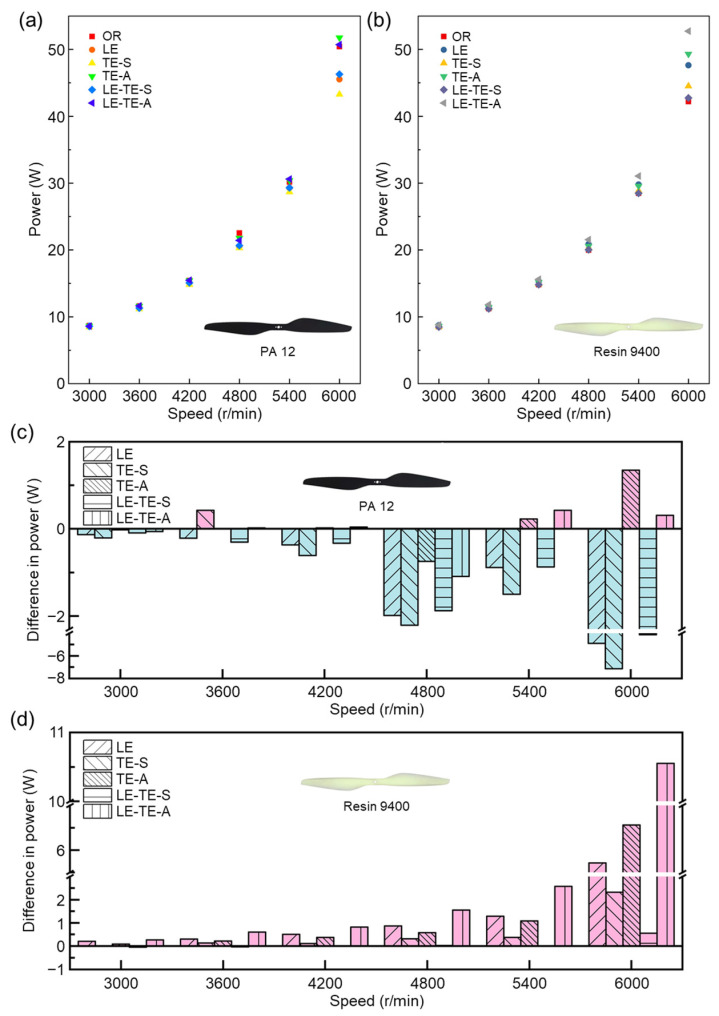
Power comparison of different rotors at various speeds. (**a**) Power consumption of bionic rotors with PA 12; (**b**) Power consumption of bionic rotors with Resin 9400; (**c**) Comparison of power consumption between bionic rotors with PA 12; (**d**) Comparison of power consumption between bionic rotors with Resin 9400. The power consumption of the OR rotor was taken as a reference value (0 W). Under the same conditions, the increase in power was positive and the decrease in power was negative.

Power is another key indicator to evaluate the aerodynamic performance of bionic rotors from energy aspect. In this section, the adjustable power supply outputs a stable voltage (*U*), and different currents (*I*) will be displayed under different rotation speeds. The power (*P*) is the product of the voltage and the current. Thus, powers of bionic rotors at different speeds were explored in detail (Figure 10). For LE-TE-A with Resin 9400, it can provide a significant increase in thrust, while the power reduction was not obvious (only 5.4% at 3600 rpm). It was understandable that air drag increased dramatically with thrust increasing during rotation, which in turn consumes additional power [39]. It can be found that the difference in power consumption was more obvious at high speed. At 6000 rpm, the power can be reduced by a maximum of 14.16% (TE-S with PA 12).

**Figure 11:**
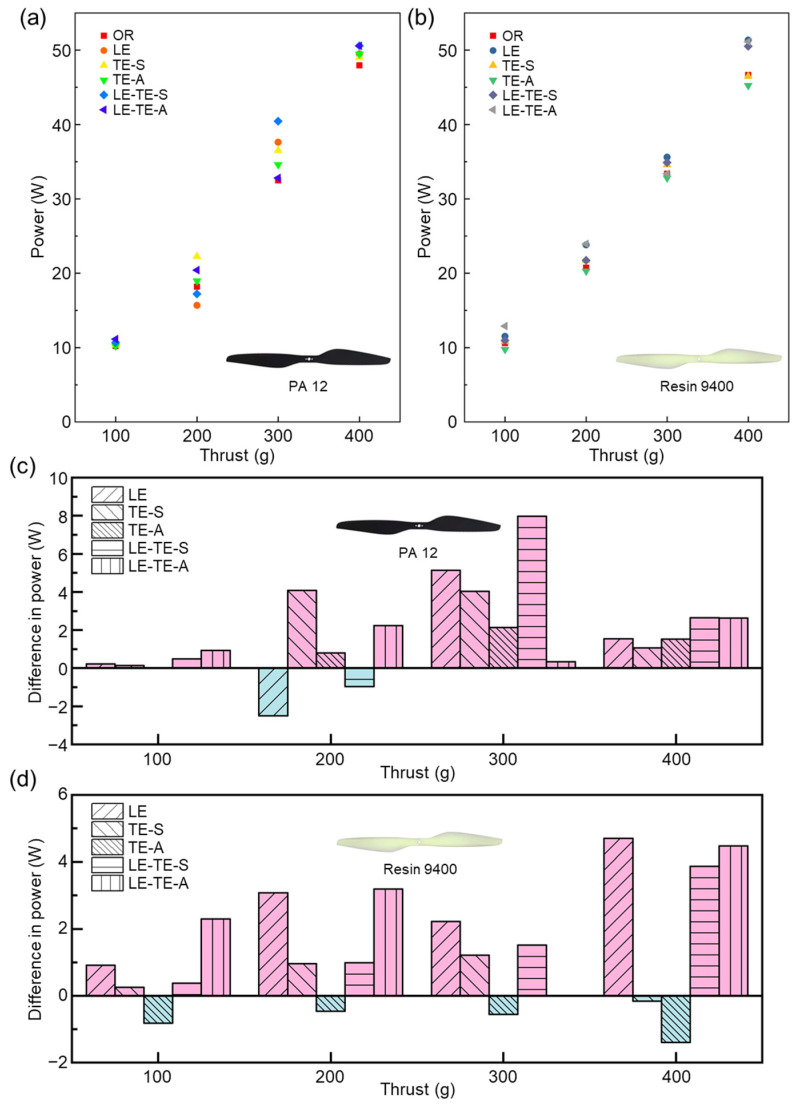
Power–thrust curves of different rotors. (**a**) Bionic rotors with PA 12; (**b**) Bionic rotors with Resin 9400; (**c**) Comparison of power consumption between bionic rotors with PA 12 under different thrusts; (**d**) Comparison of power consumption between bionic rotors with Resin 9400 under different thrusts. The power consumption of the OR rotor was taken as a reference value of (0 W). Under the same thrust, the increase in power was positive and the decrease in power was negative.

To further evaluate the aerodynamic performance of different bionic rotors, it is meaningful to compare their power consumption at the same generated thrust (Figure 11). Most obviously, when compared with OR with Resin 9400, the power consumption of TE-A with Resin 9400 was reduced for the thrust range from 100–400 g. It indicated that bionic trailing edge by AMD was effective for energy conservation. In addition, the power–thrust relationship demonstrated a nearly linear trend, which provided strong evidence of load stability of bionic rotors.

In terms of materials, bionic rotors with PA 12 suffered from aerodynamic performance degradation at high speeds (Figure 11), which could be due to the intrinsic toughness property of PA 12. Besides, bionic leading edge with sinusoidal structure (LE) showed thrust reduction as a result of effective area decreases and the modified flow field around the rotor surface. On one hand, the sinusoidal structure increased the contact area with the air during the rotor rotated, which increased the friction drag and increased the power consumption. On the other hand, serious separation of flow field around the sinusoidal structure may generate turbulent flow, which also increases the power consumption. In contrast, the existence of bionic serrated structure can effectively improve turbulent flow field near the trailing edge, thereby improve thrust [40].

### 3.3. Noise Reduction

**Figure 12:**
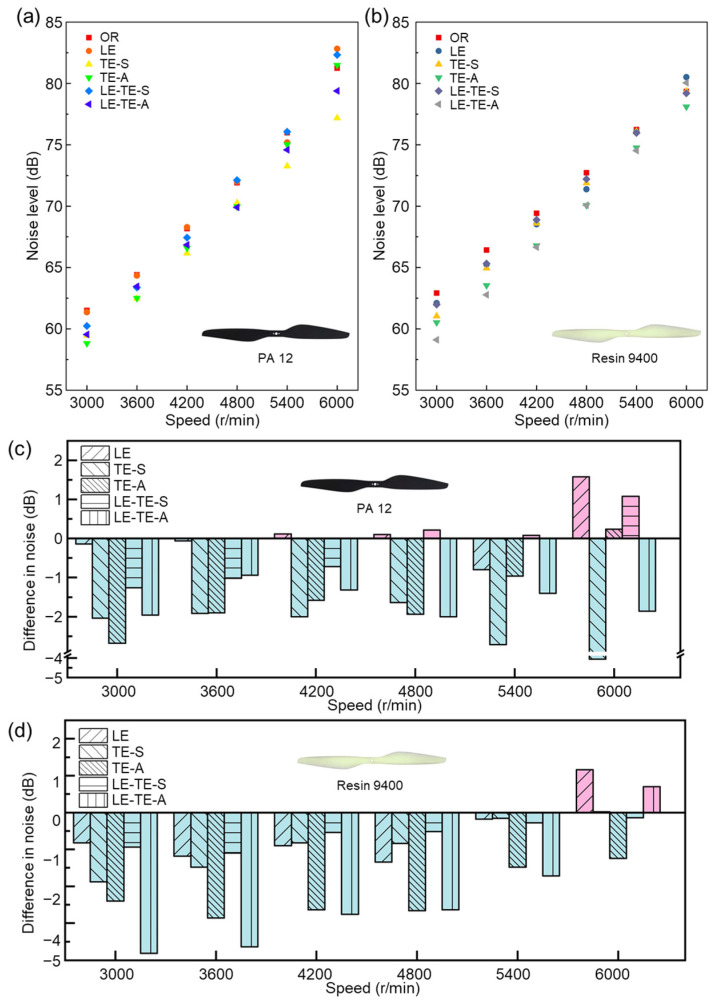
Comparison of noise level of different rotors. (**a**) Noise level of bionic rotors with PA 12; (**b**) Noise level of bionic rotors with Resin 9400; (**c**) Comparison of noise level between bionic rotors with PA 12; (**d**) Comparison of noise level between bionic rotors with Resin 9400. The noise level of the OR rotor was taken as a reference value (0 dB). Under the same conditions, the increase in noise level was positive and the decrease in noise level was negative.

Aerodynamic noise from rotor mainly arose from flow pressure disturbance on the rotor surface. To evaluate the acoustic performance of bionic rotors, noise levels at different speeds were investigated and analyzed (Figure 12). Interestingly, when compared with CF rotor at the same speed, bionic rotors with PA 12 demonstrated significant noise reduction effect in a relatively broad speed range (3000–5400 rpm) (Figure 12a). The maximal noise reduction (*NR*_max_ = *N*_Reference_ − *N*_Bionic_, *N*_Reference_ = *N*_CF_) can reach 8.9 dB (TE-S) at 3600 rpm. However, when speed reached relatively high speed (6000 rpm), the noise level of partial bionic rotors with PA 12 (LE, LE-TE-S and TE-A) climbed over 80 dB, which was slightly higher than that of the CF rotor. Surprisingly, the noise level of all the bionic rotors with Resin 9400 was much smaller than that of the CF rotor within the whole speed range (3000–6000 rpm). Particularly, for the bionic rotor LE-TE-A with Resin 9400, it showed the most significant advantage on noise reduction (Figure 12b). In terms of printing material factor, the experimental results indicated that PA 12 seemed to be superior in noise reduction than Resin 9400. The reason could be attributed to the fact that PA 12, as a ductile material, can absorb slight vibrations that arise from rotor rotation, thereby reducing noise to a certain extent.

More importantly, no matter which kind of material (PA 12 or Resin 9400) was used in 3D printing, all the rotors with bionic edges demonstrated a considerable noise reduction effect when compared with OR (Figure 12c,d). It also should be noted that noise reduction effect becomes less notable as rotation speed increases gradually. For example, when compared with OR, the *NR*_max_ (*N*_Reference_ = *N*_OR_) of bionic rotor (LE-TE-A) can reach 6.07% (3.82 dB) at 3000 rpm, while the value dropped to −0.7 dB at 6000 rpm. Without regard to printing materials, it can be found that the sinusoidal structure of bionic leading edge could be regard as a vortex generator. It affected the pressure difference between the upper and lower rotor surfaces to increase vortex, which led to more aerodynamic noise [41,42]. When compared among all the bionic rotors, the noise level of LE rotor was the highest. In contrast, the serrated structure of bionic trailing edge can smoothly decompose the airflow at the rotor tail. It effectively suppressed the generation of large eddy currents, thereby reducing noise levels from the source [43,44,45].

### 3.4. Noise Directivity

**Figure 13:**
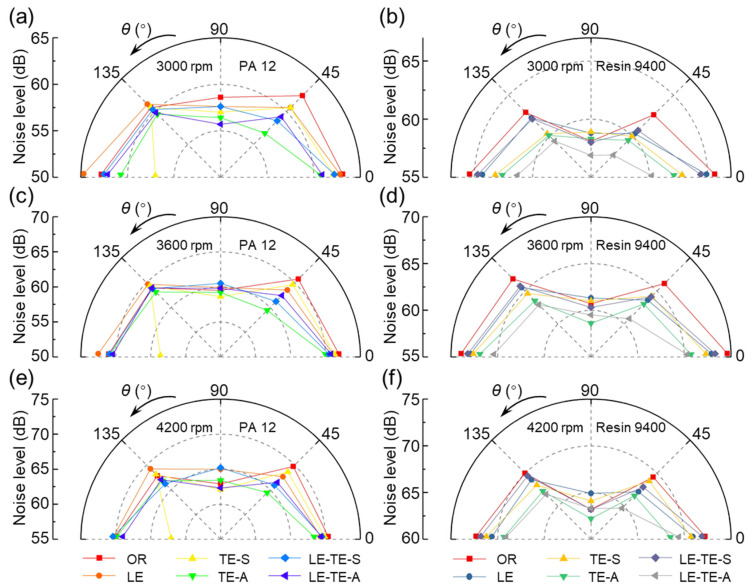
Noise directivity distribution of different rotors at 3000–4200 rpm. (**a**,**c**,**e**) Rotors with PA 12 at 3000 rpm, 3600 rpm, and 4200 rpm. (**b**,**d**,**f**) Rotors with Resin 9400 at 3000 rpm, 3600 rpm, and 4200 rpm.

**Figure 14:**
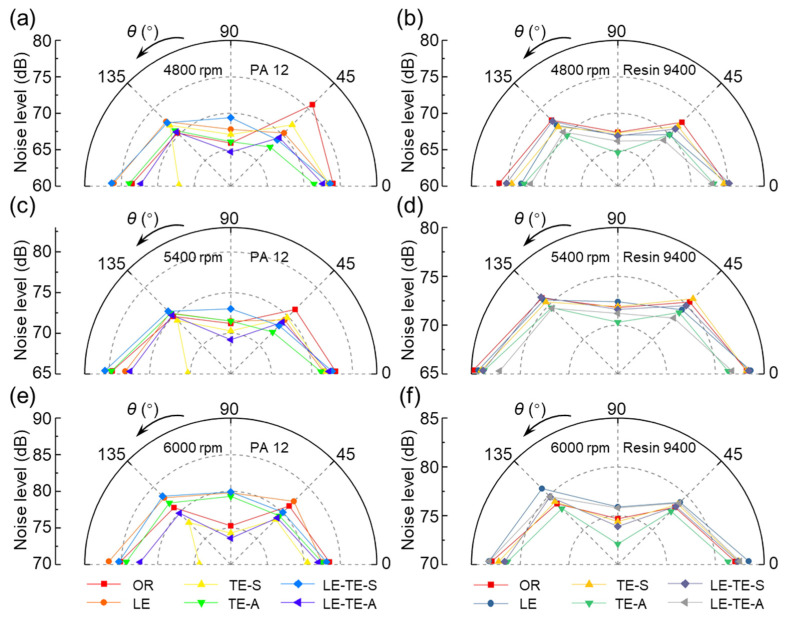
Noise directivity distribution of different rotors at 4800–6000 rpm. (**a**,**c**,**e**) Rotors with PA 12 at 4800 rpm, 5400 rpm, and 6000 rpm. (**b**,**d**,**f**) Rotors with Resin 9400 at 4800 rpm, 5400 rpm, and 6000 rpm.

In the direction perpendicular to the rotation plane, the noise generated at different positions was asymmetric. Thus, it is necessary to study the noise directivity and corresponding noise level. The noise directivities of different rotors at various speed ranging from 3000–6000 rpm were obtained (Figure 13 and Figure 14). It can be concluded that no matter what rotation speed, the noise levels of CF rotor reached an extreme point at 0° (*N*_max_) and 90° (*N*_min_), respectively. As for the OR with PA 12 and Resin 9400, the noise level at 90° was also the minimum, but the noise at 180° was similar to 0°. Particularly, some even exceed the noise level at 0° and become the highest level (OR with PA 12 at 5400 rpm and 6000 rpm, OR with Resin 9400 at 4200–6000 rpm). Therefore, the printing materials of rotors have a certain influence on the noise directivity.

Moreover, the TE-S rotor with PA 12 had a prominent noise reduction effect at 180°. It was an exciting and meaningful finding because the noise generated under the rotor directly affected the crew when the UAV was working. Compared with the OR with PA 12 rotor, the noise level of TE-S at 180° can be reduced by a maximum of 12.69% (10.8 dB, 6000 rpm). The trailing edge with serrated structure by SMD can generate a strong flow vortex to suppress the phenomenon of spanwise vortex shedding, thereby reducing noise levels. In addition, for the LE-TE-A rotor with Resin 9400 at the broad range of 3000–5400 rpm, the noise level with directivity range, from 0° to 180°, was smaller than that of the OR with Resin 9400. This situation was excluded at 6000 rpm, where the noise level became louder in all directions.

## 4. Conclusions

In summary, taking inspiration from the excellent flight characteristics of owl, five bionic rotors, including LE, TE-S, TE-A, LE-TE-S, and LE-TE-A, were designed and manufactured by 3D printing method with PA 12 and Resin 9400, respectively. OR rotor model was obtained as a control from a commercial CF rotor by a reverse inverse engineering method. Experimental study and data analysis on these rotors were carefully performed to investigate their aerodynamic and acoustical behaviors. In order to compare the effects of different bionic edge designs on rotor performance intuitively, the corresponding test results were summarized in Table 6 and Table 7. The main conclusions of this work were also drawn as follows:

(1) For aerodynamic performance, the thrust of LE rotor was reduced and the power consumption was increased, which led to a significant reduction on aerodynamic performance. This was due to the obvious decrease of effective lifting area by sinusoidal structure on leading edge. In addition, the separation of flow field near the leading edge also affected its thrust. Moreover, windward surface was formed when sinusoidal structure was constructed by removing the leading edge. It dramatically increased air friction on the windward surface, thus increasing power consumption. In contrast, for the serrated structure on the trailing edge, due to its small volume without noticeably damaging original airfoil, the manufacture method (SMD and AMD) had little influence on the effective lifting area. Interestingly, the serrated structure can improve the turbulent flow field near the trailing edge, so an enhancive thrust can also be obtained. Even the sinusoidal structure existed on the leading edge at the same time (LE-TE-A rotor with Resin 9400). Comparing the power consumed to generate the same thrust, it also confirmed that rotors with TE-A had better aerodynamic performance.

(2) In terms of acoustic performance, all bionic rotors with Resin 9400 showed decaying noise reduction effect with speed increasing. The serrated structure can smoothly decompose airflow at the rotor tail and effectively suppress the generation of large eddy currents, thereby reducing noise from the source. In contrast, the sinusoidal structure generated vortices to affect the pressure difference between the upper and lower surfaces of the rotor, which led to increased noise. In addition, printing materials also had an impact on the noise level. PA 12 with better toughness can absorb the slight tremor, resulting in less noise.

(3) For the two selected printing materials, PA 12 material had better toughness than Resin 9400. Thus, the bionic edge with PA 12 becomes soft in the thin area during the printing process, which caused possible deformation at high speed and resulted in thrust decrease as well as power consumption increase. Moreover, the PA 12 with good toughness can absorb partial vibration caused by high-speed rotation, thereby reducing the noise. Although Resin 9400 was rigid after printing, it was not strong enough to break at high speed (above 6000 rpm).

(4) Since the aerodynamic and acoustic performance of bionic rotors were strongly related to rotational speed, it was extremely important to design bionic rotors with sufficient mechanical properties to meet the rotational speed requirement. Therefore, appropriate material selection may further improve the aerodynamic and acoustic performance of bionic rotors. Thus, before rotor design in the future, material mechanical properties and processing methods should be comprehensively considered.

## Figures and Tables

**Table 1:** Parameters of different rotors models with bionic edges designs.

Rotor Type	Parameter	Relation and Value	
LE	Amplitude (*A*)	*A* = 0.1*C*_0_	2.23 mm
	Wavelength (*B*)	*B* = *C*_0_	22.3 mm
TE-S	Height (*a*)	*a* = 0.1*C*_0_	2.23 mm
	Width (*b*)	*b* = 32 *C*_0_	1.93 mm
TE-A	Height (*α*)	*α* = 0.1*C*_0_	2.23 mm
	Width (*β*)	*β* = 32 *C*_0_	1.93 mm

**Table 2:** The overall volume of different rotor models.

Rotor Type	LE	TE-S	TE-A	LE-TE-S	LE-TE-A	OR
Volume (×10^4^ mm^3^)	1.364	1.385	1.407	1.356	1.378	1.393

**Table 3:** Material parameters of PA 12 and Resin 9400.

Materials	Tensile Strength (MPa)	Breaking Strength (MPa)	Bending Strength (MPa)	Flexural Modulus (MPa)	Impact Strength (J/cm^2^)
PA 12	50	70	80	2800	23
Resin 9400	47	33.4	67	2178	27

**Table 4:** Weight comparison of 3D printing rotors with PA 12, Resin 9400, and CFRP. Unit: g.

Materials	LE	TE-S	TE-A	LE-TE-S	LE-TE-A	OR
PA 12	13.3 ± 0.5	13.7 ± 0.4	13.9 ± 0.4	13.2 ± 0.7	13.4 ± 0.4	13.8 ± 0.7
Resin 9400	15.7 ± 0.7	15.8 ± 1.0	16.1 ± 0.8	15.0 ± 0.5	15.8 ± 0.7	15.9 ± 1.0

**Table 5:** The average background noise of different motor speeds in the open space.

**Speed (rpm)**	3000	3600	4200	4800	5400	6000
**Average Noise Level (dB)**	53.7	54.7	59.0	61.2	64.4	61.1

**Table 6:** Comparison on aerodynamic and acoustic performance of different rotors with PA 12 (rounding to the one decimal place).

Performance	Speed (rpm)	OR	LE	TE-S	TE-A	LE-TE-S	LE-TE-A
Thrust (g)	3000	78.6	75.2	73.4	77.4	73.2	77.6
	3600	113.6	104.2	116.8	119.0	104.4	107.0
	4200	159.0	158.6	155.6	152.6	157.0	148.2
	4800	218.2	188.8	199.6	216.4	181.6	201.8
	5600	273.0	239.2	253.0	268.0	237.2	277.8
	6000	418.2	378.2	366.6	433.4	374.8	415.0
Power (W)	3000	8.7	8.5	8.5	8.6	8.6	8.6
	3600	11.6	11.4	11.2	11.6	11.3	11.6
	4200	15.4	15.1	14.8	15.4	15.1	15.5
	4800	22.5	20.5	20.3	21.8	20.7	21.4
	5600	30.2	29.3	28.7	30.4	29.3	30.6
	6000	50.4	45.5	43.3	51.8	46.3	50.7
Noise level (dB)	3000	61.5	61.4	59.5	58.8	60.2	59.5
	3600	64.4	64.3	62.5	62.5	63.4	63.5
	4200	68.2	68.3	66.2	66.6	67.4	66.8
	4800	71.9	72.0	70.3	70.0	72.1	69.9
	5600	76.0	75.2	73.3	75.0	76.1	74.6
	6000	81.2	82.8	77.2	81.5	82.3	79.4

**Table 7:** Comparison on aerodynamic and acoustic performance of different rotors with Resin 9400 (rounding to the one decimal place).

Performance	Speed (rpm)	OR	LE	TE-S	TE-A	LE-TE-S	LE-TE-A
Thrust (g)	3000	79.8	76.0	76.6	78.0	75.8	82.0
	3600	107.0	105.4	107.2	113.2	103.0	112.0
	4200	139.0	141.0	143.4	172.2	134.8	158.0
	4800	188.2	184.6	189.2	208.0	176.8	191.4
	5600	250.2	243.6	254.8	270.0	248.0	274.2
	6000	365.8	347.0	374.2	412.4	344.6	416.4
Power (W)	3000	8.5	8.7	8.5	8.6	8.5	8.8
	3600	11.2	11.5	11.4	11.4	11.2	11.8
	4200	14.8	15.3	14.9	15.2	14.8	15.6
	4800	20.0	20.9	20.3	20.6	20.0	21.5
	5600	28.5	29.8	28.9	29.6	28.5	31.1
	6000	42.2	47.6	44.5	49.3	42.7	52.7
Noise level (dB)	3000	62.9	62.1	61.0	60.5	62.0	59.1
	3600	66.4	65.2	64.9	63.5	65.3	62.8
	4200	69.4	68.5	68.6	66.8	68.9	66.7
	4800	72.7	71.4	71.9	70.1	72.2	10.1
	5600	76.2	76.1	76.1	74.8	76.0	74.5
	6000	79.3	80.5	79.4	78.1	79.2	80.0

## Data Availability

Not applicable.
